# Active control of micrometer plasmon propagation in suspended graphene

**DOI:** 10.1038/s41467-022-28786-8

**Published:** 2022-03-18

**Authors:** Hai Hu, Renwen Yu, Hanchao Teng, Debo Hu, Na Chen, Yunpeng Qu, Xiaoxia Yang, Xinzhong Chen, A. S. McLeod, Pablo Alonso-González, Xiangdong Guo, Chi Li, Ziheng Yao, Zhenjun Li, Jianing Chen, Zhipei Sun, Mengkun Liu, F. Javier García de Abajo, Qing Dai

**Affiliations:** 1 CAS Key Laboratory of Nanophotonic Materials and Devices, CAS Key Laboratory of Standardization and Measurement for Nanotechnology, CAS Center for Excellence in Nanoscience, National Center for Nanoscience and Technology, 100190 Beijing, China; 2 Center of Materials Science and Optoelectronics Engineering, University of Chinese Academy of Sciences, 100049 Beijing, China; 3 ICFO-Institut de Ciencies Fotoniques, The Barcelona Institute of Science and Technology, Castelldefels, 08860 Barcelona, Spain; 4 ICREA-Institució Catalana de Recerca i Estudis Avançats, Passeig Lluís Companys 23, 08010 Barcelona, Spain; 5 Department of Physics and Astronomy, Stony Brook University, Stony Brook, New York, NY 11794 USA; 6 Department of Physics, Columbia University, New York, NY USA; 7 Departamento de Física, Universidad de Oviedo, Oviedo, Spain; 8 The Institute of Physics, Chinese Academy of Sciences, P.O. Box 603, Beijing, China; 9 Department of Electronics and Nanoengineering Aalto University Tietotie 3, FI-02150 Espoo, Finland; 10 QTF Centre of Excellence Department of Applied Physics Aalto University FI-00076 Aalto, Espoo, Finland; 11 Present Address: Department of Electrical Engineering, Ginzton Laboratory, Stanford University, Stanford, CA 94305 USA

**Keywords:** Graphene, Nanophotonics and plasmonics

## Abstract

Due to the two-dimensional character of graphene, the plasmons sustained by this material have been invariably studied in supported samples so far. The substrate provides stability for graphene but often causes undesired interactions (such as dielectric losses, phonon hybridization, and impurity scattering) that compromise the quality and limit the intrinsic flexibility of graphene plasmons. Here, we demonstrate the visualization of plasmons in suspended graphene at room temperature, exhibiting high-quality factor *Q*~33 and long propagation length > 3 μm. We introduce the graphene suspension height as an effective plasmonic tuning knob that enables in situ change of the dielectric environment and substantially modulates the plasmon wavelength, propagation length, and group velocity. Such active control of micrometer plasmon propagation facilitates near-unity-order modulation of nanoscale energy flow that serves as a plasmonic switch with an on-off ratio above 14. The suspended graphene plasmons possess long propagation length, high tunability, and controllable energy transmission simultaneously, opening up broad horizons for application in nano-photonic devices.

## Abstract (web summary)

Graphene plasmons hold potential for infrared optoelectronic devices, but the interaction with the substrate often degrades their quality. Here, the authors report the characterization of plasmons in suspended graphene with tunable suspension height, showing enhanced quality factors and propagation lengths at room temperature.

## Introduction

Graphene plasmons (GPs), the collective oscillations of Dirac-fermion electrons in doped graphene, enable subwavelength light confinement in the infrared (IR) and terahertz frequency domains^1–3^, offering an ideal platform to support high-speed^4^, low-damping^5,6^, actively-tunable energy transport at the nanoscale^7,8^. Importantly, GP properties can be dynamically tuned by changing the graphene Fermi energy through electrical gating, which finds applications ranging from light modulation to light detection and sensing^9–11^. In addition, extensive efforts have been devoted to creating passive graphene components for plasmonic circuitry, such as waveguides, resonators, and couplers^12^. However, the current state-of-the-art of GPs is far from real-world applications due to their short propagation length, limited tunability, and lack of energy flow controllability. This is because the performance of GPs is limited by a supporting substrate underneath graphene, which brings about extrinsic damping pathways, including dielectric losses, phonon hybridization, and impurity scattering. Such extrinsic damping comes mainly from the natural IR phonons that reside in the chosen substrates, such as SiO_2_ and BN^5,6^. Even at cryogenic temperatures, dielectric losses from the environment still contribute a significant damping that cannot be overcome by improving the intrinsic properties of graphene^13^. The dielectric permittivity and phonon hybridization also restrict the operating frequency bandwidth of GPs. Besides, when plasmons propagate across different substrates, electromagnetic energy is affected by the dramatic change of the local dielectric environment, thereby causing reflection, transmission, and radiative out-coupling associated with the plasmonic impedance mismatch between the two different dielectric environments^14–17^. Therefore, the design of a dielectric environment to address the aforementioned challenges in one system is essential to bring GPs close to real photonic devices.

Suspended graphene with very high carrier mobility approaching ballistic transport and long carrier relaxation time^18,19^ serves as an ideal platform to avoid dielectric losses from the substrate^20^. Notably, suspended graphene structures have been widely explored in photodetectors, ultrafast photocurrents and terahertz generation^21^, visible light emission^22^, nanomechanical resonators^23^, and thermal transport devices^24^. Here, we demonstrate that a remarkable improvement in the quality factor and propagation length of GPs can be achieved in suitably designed suspended graphene plasmonic structures. Furthermore, a broad tunability of the plasmon dispersion, propagation length, phase and group velocities, and energy flow is demonstrated by merely in situ adjusting the suspension height, leading to a change of dielectric environment that is difficult to achieve in substrate-supported plasmonic structures. Based on the active control of plasmon micrometer propagation, we implement an effective switch for near-unity-order control of plasmonic energy.

## Results

### Plasmonic response and dispersion in suspended graphene

**Fig. 1:**
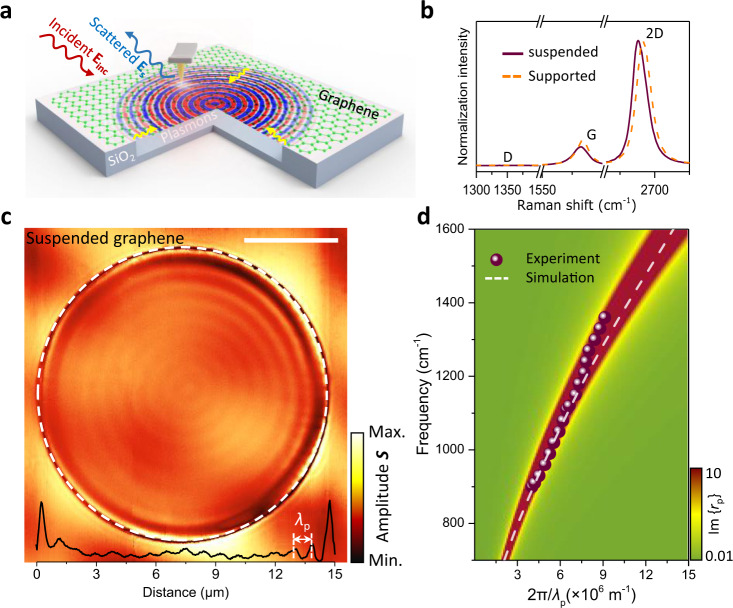
Plasmonic response and intrinsic dispersion in suspended graphene structures. **a** Sketch of experimental arrangement for launching and detecting plasmons propagating in suspended graphene. The tip and sample are illuminated with a focused infrared light wave (with wavelength λ_0_ and field **E**_inc_), which can induce dipoles around the hole edge, thus efficiently exciting the graphene plasmons (yellow arrows). The tip subsequently scatters the plasmons, and a distant detector measures the scattered field **E**_S_. Note that we use defocus processing to enlarge the spot size to ~30 microns and enhance the surrounding edge launching efficiency further. **b** Raman spectra of suspended and SiO_2_-supported regions of high-quality undoped graphene. **c** Near-field optical signal at λ_0_ = 10.87 μm (920 cm^−1^) light wavelength from a two-dimensional scan across the suspended graphene area with a circular diameter Φ = 15 μm and a suspension height ~450 nm. The interface between suspended and supported graphene regions is marked with a dashed white circle. The black curve at the bottom shows the near-field profile along with a horizontal cut through the center of the circular hole. The graphene Fermi energy is *E*_F_ ~ 0.68 eV. The scale bar indicates 5 μm. *λ*_p_ represents the plasmon wavelength. **d** Experimentally measured (symbols, from a device with Φ = 2.5 µm and *E*_F_ ~ 0.9 eV, shown in Supplementary Fig. 4) and simulated (white dashed curve, details in Methods) dispersion of suspended GPs. The loss function Im{*r*_p_(q, ω)}, calculated from the reflection coefficient *r*_p_ in the random-phase approximation (RPA, details in Methods), is shown as a pseudo-color-plot background.

As schematically shown in Fig. 1a, we deposit mechanically exfoliated graphene flakes onto a SiO_2_/Si substrate perforated with circular dimples of different diameters in the 30 nm–50 μm range (see also Supplementary Fig. 1). Our fabrication procedure (details in Methods) avoids contamination by chemical agents, as well as mechanical damage from the surface tension of the liquid to maintain intact the intrinsic properties of graphene during the transfer process. Circular dimples are mainly used to prevent wrinkles and achieve better control of the suspended structure. The holes underneath suspended graphene are filled with N_2_ gas to avoid the collapse of the carbon monolayer and change its distance to the substrate by controlling the gas pressure. The samples are then sealed in a chamber with NO_2_ gas (concentrations are varied between 25 and 75% in N_2_ atmosphere) for several hours to achieve a high doping level by surface adsorption of gas molecules, which act as electron acceptors^25,26^. The monolayer thickness of the graphene used in this work is confirmed by the Lorentzian profiles of the 2D peak in its Raman spectra (Fig. 1b and Supplementary Fig. 2), while the absence of a defect-induced D band (1350 cm^−1^) indicates a high quality of the samples. By comparison, the G peak of suspended graphene redshifts by 2.1 cm^−1^, indicating unnoticeable strain (only 0.014%) induced in the carbon monolayer by our suspension structures. Besides, the Raman ratio *I*_2D_/*I*_G_ decreases significantly from 5 in suspended graphene to 3 in supported graphene due to the scattering of graphene electrons caused by charged impurities from the SiO_2_ substrate. The doping level can be directly controlled through the gas concentration and doping time (Supplementary Fig. 2), which can shift the graphene Fermi energy *E*_F_ up to ~0.9 eV. The resulting Fermi energy is much higher than that provided by the commonly employed electrostatic gating (typically changing the graphene carrier density by up to ~4 × 10^12^ cm^−2^, corresponding to a Fermi energy shift of ~0.3 eV from the neutrality point). We find that plasmons remain almost unchanged for several hours and gradually degrade after a few days due to the desorption of NO_2_ gas molecules. Interestingly, the plasmons can be effectively turned off by thermal enhancement of desorption in our experiments (e.g., heating the sample at 100 °C for 5 min in N_2_ atmosphere).

We use a scattering-type scanning near-field optical microscope (s-SNOM) with a tunable quantum cascade laser operating in the 892–1600 cm^−1^ range to image propagating plasmons in graphene. The IR light beam is focused on a metallic atomic force microscope (AFM) cantilever probe tip (Fig. 1a). The backscattered near-field optical signal can be recorded simultaneously with the topography. The broad scan range of s-SNOM (up to 50 × 50 μm^2^) is critical for the characterization of long-distance propagation. In previous studies, it was well established that plasmon polaritons manifest as a periodic modulation (fringes) of the observed near-field signal as a function of the tip position relative to graphene edges and other surface features^2,3^. Furthermore, s-SNOM can distinguish tip- and edge-launched plasmons^9^, thus providing a valuable platform for characterizing plasmon propagation, reflection, and transmission^14,15^.

We first explore plasmon polaritons propagating on a large-area suspended graphene sample (*E*_F_ ≈ 0.68 eV) with an IR laser operating at λ_0_ = 10.87 μm (920 cm^−1^). In the near-field nanoscopy image shown in Fig. 1c, plasmonic fringes remarkably cover the entire large suspended graphene area (with a circular diameter Φ = 15 μm). The corresponding optical and topography micrographs are shown in Supplementary Fig. 1. The wavelength *λ*_p_ of the suspended GPs is calculated to be 938 nm from analytical RPA theory^27^ and numerical simulations^28^, a value that agrees well with the experimentally observed distance between two adjacent fringes in Fig. 1c. As a salient feature of suspended GPs, we remark that their wavelength is much longer than that of graphene samples supported by substrates^2,3,5^. Besides, inspection of Fig. 1c reveals that the plasmonic oscillations extend 7.5 μm beyond the edge of the dimple; the propagation length is limited by the sample size and far exceeding previous records on any other substrates at room temperature^2,3,5^. In contrast, the nanoscopy images of suspended graphene without doping are featureless (Supplementary Fig. 3).

To investigate plasmon dispersion as a function of the wave vector *q* and photon energy *ω*, a smaller suspended structure (diameter Φ = 2.5 μm) is chosen to obtain more accurately measured near-field amplitude images. Fringe patterns can be observed with a periodicity corresponding to *λ*_p_/2, which is confirmed by our electromagnetic simulations (Supplementary Figs. 4, 5). The plasmon dispersion relation extracted from experimental measurements (dots in Fig. 1d) is plotted and compared with RPA calculations (pseudo-color-plot background)^27^. Fringes of two distinct periodicities (*λ*_p_ and *λ*_p_/2) appear in Fig. 1c and Supplementary Fig. 4, respectively. The *λ*_p_/2-period fringes can be assigned to tip-launched plasmons reflected at the edge of the suspended area and subsequently out-coupled to radiation by the tip^2^. The plasmon edge-reflection is caused by the mismatch of capacitive coupling across dielectric interfaces, mainly determined by the difference in the wave vectors of plasmons between substrate-supported and suspended regions. The *λ*_p_-period fringes can also be produced by edge-launched plasmons propagating to the tip and being out-coupled^9^. We note that the efficiency of plasmon excitation at the edges depends on the combined effect of dielectric polarization^29^ and the size and shape of the hole edge (Supplementary Note 1)^30^.

With the ultrahigh doping levels enabled by suspended graphene, we can access a wide range of plasmon wavelengths, ranging from 850 to 1560 nm with different excitation frequencies in our suspended graphene sample. Moreover, the plasmon dispersion relation shown in Fig. 1d presents a relatively steep slope, eventually covering excitations by light frequencies up to 1400 cm^−1^. The operational frequencies are limited by the Fermi energies and are generally reaching up to 1000 cm^−1^ by electrical gating. Besides, suspended graphene can be easily tailored into different nanoresonator shapes and sizes by engineering the shape of the hole carved in the substrate rather than patterning graphene itself, thus offering a unique opportunity to manipulate plasmonic propagation and localization. For example, the hole size in the substrate can be reduced down to <50 nm, providing robust on-demand hotspots that are appealing for numerous applications in the field of nanophotonics (Supplementary Fig. 6)^12^.

### Long-distance propagation of suspended graphene plasmons

**Fig. 2:**
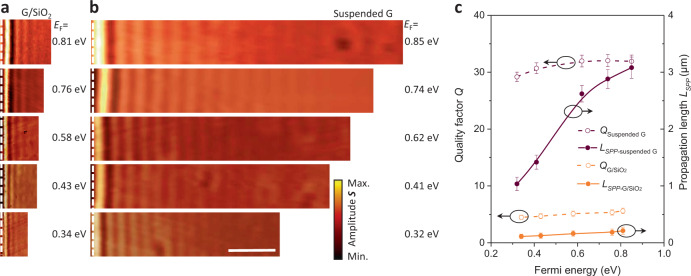
High-quality factor and long-distance propagation of suspended GPs. **a**, **b** Measured near-field amplitude images of SiO_2_-supported graphene (**a**) and suspended graphene (suspension height ~450 nm) (**b**) near the edge (white dashed line) for different graphene Fermi energies and fixed illumination wavelength λ_0_ = 10.87 μm (920 cm^−1^). The scale bar indicates 3 μm. **c** Quality factor *Q* (left axis) and propagation length *L*_SPP_ (right axis) of suspended (maroon curves) and SiO_2_-supported (orange curves) GPs as a function of Fermi energy. Symbols are obtained from experimental data, while curves are a guide to the eye. Error bars indicate 95% confidence intervals.

While in Fig. 1c we have demonstrated a considerable propagation length of suspended GPs, in Fig. 2a, b we compare the near-field amplitude images of plasmon propagation in suspended and SiO_2_-supported graphene with similar Fermi energies. The near-field images in Fig. 2b are flanked by the boundary (indicated by the white dashed lines) of a circular suspended region with a diameter of 35 μm and a suspension height of ~450 nm. To quantify the improvement brought about by suspended graphene, we need a direct comparison of the quality factor *Q*. In momentum space, the complex, frequency-dependent plasmon wave vector is given by^2,13^ ${q}_{p}\left(\omega \right)={q}_{1}\left(\omega \right)+i{q}_{2}\left(\omega \right)=\frac{i\kappa \omega }{2\pi \sigma (\omega )}$, where subindices 1 and 2 indicate the real and imaginary parts, respectively. Here, $\sigma (\omega )$ is the graphene surface conductivity and *κ* is the effective dielectric function of the graphene environment. The quality factor *Q* is given by ${Q}^{-1}=\frac{{q}_{2}}{{q}_{1}}\approx \frac{{\sigma }_{1}}{{\sigma }_{2}}+\frac{{\kappa }_{2}}{{\kappa }_{1}}$, under the conditions $\left|{\sigma }_{2}\right|\gg \left|{\sigma }_{1}\right|$ and $\left|{\kappa }_{1}\right|\gg \left|{\kappa }_{2}\right|$, which are well satisfied in this study. Also, the intrinsic plasmon propagation length (for 1/*e* attenuation of intensity) can be expressed as ${L}_{{SPP}}=\frac{1}{{2q}_{2}}=\frac{1}{4\pi }{\lambda }_{p}Q$. We implement a multi-beam interference model (Supplementary Fig. 7 and Note 2) to analyze complex near-field amplitude images^31^, from which ${q}_{2}\left(\omega \right)$ can be extracted through direct real-space fitting of the near-field signal line scans (see Supplementary Fig. 8 and Note 2 for more details of the analysis method).

The extracted values of *Q* and *L*_SPP_ as a function of Fermi energy (Fig. 2c and Supplementary Fig. 9) reveal a remarkable improvement in suspended graphene samples (maroon curves) over supported ones (orange curves). The quality factor of suspended GPs varies from *Q* = 29 to *Q* = 33 when *E*_F_ is changed from 0.3 to 0.8 eV, while in SiO_2_-supported plasmons *Q* stays below 5.4. In addition, *L*_SPP_ increases from 1.0 to 3.1 μm with increasing *E*_F_ in suspended GPs, while it is limited to the 0.1–0.2 μm range in SiO_2_-supported plasmons as a result of their smaller quality factor and plasmon wavelength. In addition, the plasmon wavelengths of graphene suspended in air (dielectric constant ${{{{{{\rm{\varepsilon }}}}}}}_{air}$≈1) and supported on a SiO_2_ substrate satisfy the relation λ_suspended_/λ_supported_ = (1 + ${{{{{{\rm{\varepsilon }}}}}}}_{{{SiO}}_{2}}$)/2, where the real part of ${{{{{{\rm{\varepsilon }}}}}}}_{{{SiO}}_{2}}$ is 3.8 at 920 cm^−1^ ^32^. The improvement in the quality factor in suspended graphene is due to the reduced environmental losses brought about by the absence of a substrate (Supplementary Note 1). However, the quality factor of our suspended graphene samples does not reach the estimated intrinsic limit *Q* ~ 40–70 at room temperature^5^. We attribute this to impurity scattering produced by gas-molecule dopants, which provide an extra extrinsic decay channel^33^. A possible mechanism could be related to the exchange of momentum with the graphene crystal lattice, as NO_2_ molecules have a size comparable to the unit cell, leading to local heating and additional plasmon damping. Nevertheless, we observe that the quality factor of suspended GPs samples is always higher than that of supported GPs at room temperature.

Note that our experimentally measured value of *Q* for suspended GPs exceeds by 35% that found in previous work for plasmons on high-quality graphene encapsulated in a hexagonal BN sandwich configuration at room temperature^5^. However, the performance of propagating plasmons in a suspended sample at room temperature is still not comparable to that in encapsulated graphene at low temperature^13^. This proves that phonons exert a significant influence on the dissipation of GPs. We also note that effective electrical gating of suspended graphene could be used to suppress losses associated with impurity scattering and push *Q* further to its intrinsic limit, which is increased by 2–3 times at room temperature and one order of magnitude at low temperature.

### Active control of suspended graphene plasmons

**Fig. 3:**
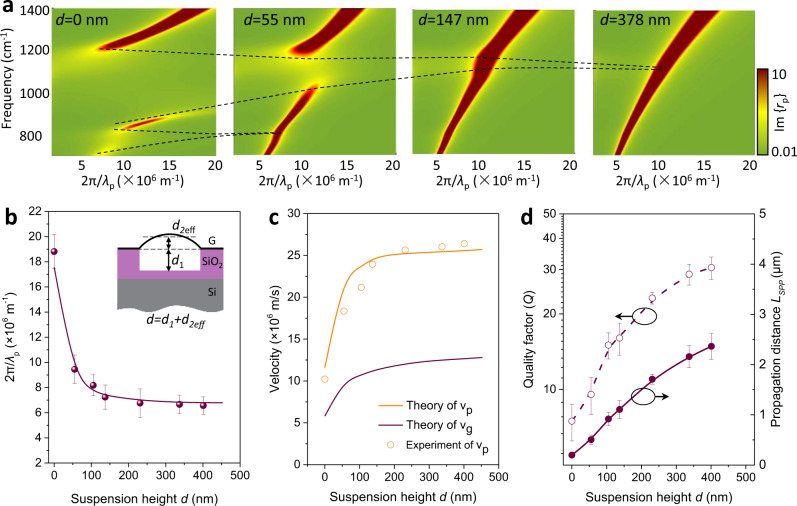
Active control of suspended graphene plasmons by changing the suspension height. **a** Calculated imaginary part of the loss function Im{*r*_p_(q, ω)} in the RPA for different suspension heights *d*, with fixed *E*_F_ = 0.65 eV and *λ*_0_ = 10.87 μm (920 cm^−1^). The black-dashed curves indicate the evolution of the energy splitting stemming from the coupling between GPs and phonon polaritons in SiO_2_. **b** Experimentally measured (symbols) and theoretically calculated (solid curve) plasmon wave vector as a function of the suspension height *d* at *λ*_0_ = 10.87 μm (920 cm^−1^). Inset: illustration of a cross-sectional side view of the suspended graphene device. *d*_*1*_ and *d*_*2eff*_ represent the depth of the dimple and the effective height of the graphene bubble, respectively. The former (*d*_*1*_) is predetermined by the fabrication process, while *d*_*2eff*_ can be varied in situ by controlling the gas pressure from beneath the substrate film. **c** Extracted phase (orange curves) and group velocity (maroon curves) as a function of *d*. Solid curves are theoretical predictions, while symbols are values determined from the experiments. **d** Quality factor *Q* (left axis) and intrinsic propagation length *L*_SPP_ (right axis) of GPs as a function of *d*. Symbols are obtained from experimental data, while the curves are a guide to the eye. Error bars correspond to different line profiles in one scan image.

The elimination of interactions from the substrate renders a pure plasmonic environment, providing a unique platform for manipulating the out-of-plane interaction between intrinsic GPs and the dielectric environment. In Fig. 3a, we show the calculated plasmon dispersion with different suspension heights *d*. The height *d* between suspended graphene and the substrate is taken as the sum of the hole depth *d*_*1*_ and the height of the graphene bubble *d*_*2eff*_ relative to the substrate plane. The strength of long-range Fröhlich coupling between GPs and the optical phonons of the SiO_2_ substrate is strongest when graphene is directly placed on top of the substrate (*d* = 0), where the dispersion splits into three bands^6^. We observe that, as *d* increases from 0 to 453 nm, the coupling strength gradually weakens (black-dashed curves in Fig. 3a) and eventually disappears at *d* = 55 nm and 147 nm for the surface optical phonons at ω_1_ = 806 cm^−1^ and ω_2_ = 1168 cm^−1^, respectively. In Supplementary Fig. 10, we show the corresponding measured near-field amplitude images with increasing suspension height *d*. Note that when *d*_2_ is positive, we define an effective height to reduce the deviation caused by the parabolic shape of the suspended graphene bubble. The effective height is given by *d*_*2*eff_ = (1/*r*) ${\int }_{-r}^{r}h(x){{{{{\rm{d}}}}}}x$, where *h*(x) is obtained by fitting the height profile to a parabola. Following this approach, the plasmon wavelength can be tuned over a wide range (from 400 nm to 1.2 μm) due to its strong dependence on the permittivity of the environment. Figure 3b shows that, for different suspension heights *d*, the plasmon wave vectors extracted from the measured near-field amplitude images (symbols) agree well with the calculated results (maroon curve)^34^. Besides the significant control obtained over the plasmon wavelength by changing the suspension height, both phase (ν_P_ = ω/q_1_) and group (ν_g_ = ∂ω/∂q_1_) velocities sharply increase with *d* in the *d* < 80 nm region, and they saturate at *d* ~ 200 nm, as shown in Fig. 3c. Notably, ν_g_ can vary within a range of 0.42–2.5 × 10^7^ m/s, suggesting the possibility of broadly controlling the propagation speed of information in a graphene waveguide.

Modulation of the interaction between GPs and a substrate can also affect the plasmon quality factor. In Fig. 3d and Supplementary Fig. 10b, we show the analysis of the quality factor *Q* and propagation length *L*_*SPP*_ for different suspension heights *d*. Once graphene is detached from the substrate, the quality factor *Q* increases sharply due to the reduced coupling to dielectric losses from the SiO_2_ substrate (see detailed discussion in Supplementary Fig. 11 and Supplementary Note 3). This is consistent with the exponential decay of the electric field associated with plasmons away from the graphene plane. By computing the percentage of the near-field intensity in the proximity of graphene, we find that 65% of the mode energy (*λ*_p_ ≈ 1.5 μm at a suspension height of *d* = 453 nm) is confined within 100 nm above or below the graphene plane^11^. We observe a twofold improvement of *Q* when *d* increases to 55 nm, while a milder increase in *Q* takes place after *d* exceeds 100 nm. For the achieved maximal suspension height (*d* = 453 nm), the quality factor *Q* is enhanced by about a factor of six, and the corresponding *L*_*SPP*_ increases by one order of magnitude (from 0.25 to ~3.0 μm) compared to the values observed in supported graphene.

### Plasmonic switch based on suspended graphene plasmons

**Fig. 4:**
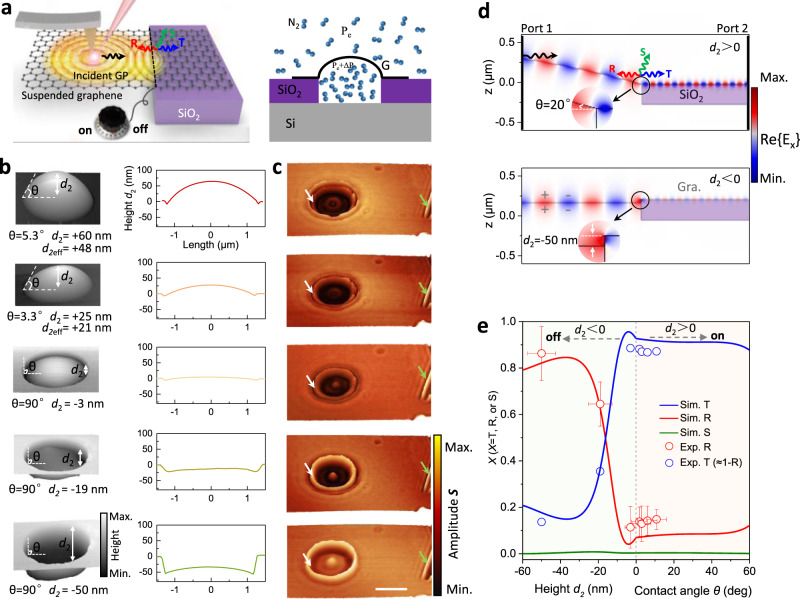
Plasmonic switch based on tunable control of GP transmission at air-dielectric substrate interfaces. **a** Schematic of the plasmonic switch. Black, red, and blue arrows represent incident, reflected, and transmitted plasmons, respectively. The green arrow stands for plasmon scattering (out-coupling) into radiation. The black-dashed line indicates the air-dielectric interface. **b** AFM topography images of suspended graphene with different heights of the graphene bubble *d*_*2*_, obtained by controlling the gas pressure for fixed hole depth *d*_*1*_ = 300 nm (see definitions in the inset of Fig. 3b). A negative value of *d*_*2*_ indicates that the graphene bubble is sunken inside the substrate dimple. We use a sample with a large value of *d*_1_ to avoid any complications in the interpretation. *θ* is the contact angle of suspended graphene and the substrate. **c** Near-field IR images of suspended graphene, taken simultaneously with the AFM topography for the corresponding suspension heights shown in (**b**). The incident light wavelength is λ_0_ = 10.87 μm (920 cm^−1^), and the graphene Fermi energy is 0.40 ± 0.03 eV. Since the entire sample is treated with the same gas concentration and doping time, we assume that the graphene has the same Fermi energy in the substrate and suspended regions. The bright fringes at the air-dielectric interface and natural graphene edge are indicated by white and green arrows, respectively. The scale bar indicates 2 µm. **d** Simulated spatial distribution of the electric field along the *x* direction as GPs propagate from the suspended region to the region supported by the SiO_2_ substrate, with *d*_2_ > 0 (upper part) and *d*_2_ < 0 (bottom part). Inset: expanded view of the boundary area. **e** Plasmon reflectance (red), transmittance (blue), and scattering (green) as a function of *d*_*2*_ and *θ*. Colored curves are numerical simulations with *E*_F_ = 0.4 eV, whereas symbols represent experimental results extracted from (**c**). The vertical dashed line and two shaded areas are used to indicate the two cases where the suspension height is positive or negative. Error bars are extracted from different line profiles in each scanned image.

Both the long-distance propagation and control of suspended GPs offer unique possibilities for studying applied aspects of graphene plasmonics. Nanoscale energy transfer and management mediated by plasmons at the interface between two media are vital for the design of nanophotonic devices^14,16,35^. In Fig. 4, we demonstrate that plasmon energy flow through the air-dielectric interface can be controlled by gradually changing the shape of the suspended graphene, serving as an effective plasmonic switch. Our experiment setup is illustrated in Fig. 4a. Indeed, we can tune the height *d*_*2*_ of a graphene bubble from −50 nm (concave shape) to +60 nm (convex) by changing the gas pressure, as revealed by AFM (Fig. 4b) and the corresponding near-field amplitude images of the circular suspended structure shown in Fig. 4c. Pronounced plasmon interference fringes at both air-dielectric interfaces (indicated by a white arrow) and natural graphene edges (indicated by a green arrow) can be observed, which suggest that plasmons can be efficiently reflected by the air-dielectric interface, besides the well-known total reflection at the natural graphene edges^14^. The reflectance coefficient can be estimated as *r* = (*S*_dielectric_-*S*_sheet1_)/(*S*_edge_-*S*_sheet2_)^14,36^, where *S*_dielectric_, *S*_edge_, *S*_sheet1_, and *S*_sheet2_ represent the near-field signals of the bright fringe at the air-dielectric interface, the bright fringe at the natural graphene edge, and from the background of suspended (*S*_sheet1_) and supported (*S*_sheet2_) graphene, respectively (Supplementary Fig. 12). Note that the diameter of the hole is 2.5 μm, which is larger than the propagation length of the graphene plasmon (*L*_spp_ = 1.4 μm) at the considered Fermi energy *E*_F_ = 0.4 eV (Fig. 2c). Therefore, the value of *S*_dielectric_ is essentially determined by the first fringe amplitude near the air-SiO_2_ interface. Since the near-field amplitude *S* tracks variations in the local electric field between tip and graphene, the plasmon energy scales as the square of *S*, and then the reflectance can be evaluated as *R* = |*r*|^2^. Figure 4d illustrates the spatial distributions of the real part of the electric field Re $\left\{{E}_{x}\right\}$ along the horizontal direction for the transmission of GPs from suspended to SiO_2_-supported graphene regions, where a plasmonic impedance mismatch between the two regions appears due to the abrupt change of substrate permittivity and graphene morphology at the interface. We also note that both the contact angle *θ* and the step height *d*_2_ influence the plasmonic impedance mismatch and further affect the plasmonic transmission (Supplementary Fig. 13).

When the suspended graphene is above the substrate surface (i.e., *d*_2_ > 0, see upper part of Fig. 4d), it is convenient to exploit the contact angle *θ* (determined by the value of *d*_2_ and the graphene morphology) to control the reflection at the interface due to a different graphene morphology compared with the situation in which *d*_2_ < 0. When *θ* gradually increases from 0 to 45°, the reflectance gradually increases from 6.5 to 8.6%. However, when the graphene is below the substrate surface in the suspended region (i.e.*, d*_2_ < 0, bottom part of Fig. 4d), it attaches to the sidewall of the dimple due to van der Waals attraction, and the contact angle *θ* stays at 90°, whereas *d*_2_ mainly determines the reflection. As *d*_*2*_ increases from −50 nm to −3 nm, the fringe contrast in the suspended graphene region gradually decreases, indicating a reduced reflectance at the air-dielectric interface. We experimentally observe that the reflectance is close to 90% when *d*_*2*_ decreases to −50 nm with *E*_F_ ≈ 0.4 eV (Fig. 4e, symbols).

The minimum reflectance is 6.5% if the graphene bubble is nearly flat (*d*_*2*_ = 0, i.e., co-planar with the substrate surface). Due to the small thickness of single-atomic-layer graphene, almost no free radiation is emitted in the scattering process under these conditions (green curve in Fig. 4e)^36^, so the maximum energy crossing the air-dielectric interface can be estimated to be 93.5% from the measured reflectance (*T* = 1-*R*)^37^. By analogy to electronic devices, we can define the ratio between the maximum and minimum energies passing across the interface as the switch ratio of plasmonic devices^38^; the observed switch ratio reaches >14 by in situ changing the shape of suspended graphene. Simultaneously, the plasmon wavelengths are very different at the two sides (λ_p_ ≈ 530 nm at the left in the suspended graphene region, and λ_p_ ≈ 170 nm at the right in the SiO_2_-supported area, as shown in Fig. 4d). Therefore, the device serves as an efficient plasmon wavelength converter.

It is worth noting that a suspended graphene electromechanical system driven by gate voltage can be used to actively control the reflection. As a thin atomic membrane with low lateral stiffness, suspended graphene can be ideal for electromechanical devices to control its morphology. Indeed, suspended graphene can be gradually sucked into the holes with a vertical displacement under a transverse electric field. The height of suspended graphene relative to the substrate can be tuned from +5 nm to −26 nm with a gate voltage varying from 0 to 30 V (Supplementary Fig. 14 and Note 4). With a continuous increase in gate voltage, the differences of height and Fermi level on both sides can be exacerbated simultaneously, further promoting the reflection of plasmons at the boundary. As shown in Supplementary Fig. 14, plasmon reflection at the dielectric boundary increases with gate voltage from 23 to 70%. Other controllable parameters, such as the graphene Fermi energy and the incident light frequency, can also offer versatile methods for tunability (Supplementary Figs. 15, 16 and Notes 5, 6).

## Discussion

To conclude, we demonstrate that high-frequency propagating plasmons (~1400 cm^−1^) can be excited in suspended monolayer graphene with an ultrahigh doping level (up to 0.9 eV) introduced through gas-molecule adsorption. Importantly, a high-quality factor (*Q* = 33) and a long propagation length (*L*_SPP_ > 3 μm) in plasmons evolving in tailored suspended graphene at room temperature are achieved by eliminating interactions with a substrate. In addition, suspended graphene provides a unique clean plasmonic platform that grants us better understanding and tuning of the interaction physics of the extrinsic environment with GPs. More precisely, by in situ changing the suspension height of graphene, we implement an efficient manipulation of the plasmon wavelength, propagation length, and phase/group velocity. We further present an effective route to fully control the transmission of plasmonic energy at the air-dielectric hole-edge interface by applying electromechanical concepts to plasmonic devices, in which the suspended graphene can be deformed by either introducing a gate voltage or varying gas pressure. Furthermore, we discuss how the deformation can be tailored to create a plasmonic switch (by gas) and transistor (by gating) to achieve near-unity-order manipulation of plasmonic energy flow. The combination of micrometer propagation and energy manipulation can be applied to design other advanced devices, such as plasmonic resonators, filters, and modulators, which are pivotal to control energy flow in ultra-compact integrated plasmonic circuits.

## Methods

### Nanofabrication of suspended GP devices

Circular holes with sizes ranging from 30 nm to 50 μm were patterned on a 300 nm SiO_2_/500 μm Si substrate using 100 kV electron-beam lithography (EBL) (Vistec 5000+ES, Germany) on ~350 nm of ZEP520A electron-beam lithography resist. The hole arrays were etched by C_4_F_8_ and SF_6_ gases (North Microelectronics, DSE200). Then, the resist layer was dissolved with butanone and the whole wafer was cleaned with isopropyl alcohol. For holes that were deeper than 300 nm, we used plasma-enhanced chemical vapor deposition to grow a 300 nm-thick layer of SiO_2_ in the hole to cover the silicon substrate. The remaining residues on the silicon oxide surface were removed by oxygen plasma cleaning with 5 Pa and 80 W for 20 min. Graphite flakes were then mechanically deposited onto the substrate. Optical microscopy was used to identify monolayer graphene sheets, and their layer numbers were further confirmed by Raman spectroscopy. To control the shape of suspended graphene, the samples were placed into the chamber with N_2_ to create a pressure difference (*ΔP*) between the inside and outside of the suspended graphene membrane. *ΔP* was utilized as a controlling load to change the suspension height based on the well-established gas diffusion method according to *ΔP* = (*P*_*a*_V_0_/(V_0_ + *V*_*s*_))-P_e_^39^, where P_a_ and P_e_ are the applied and external atmosphere pressures; and V_0_ and V_s_ are the volumes of the hole and suspended graphene bubble, respectively. A schematic diagram of the gas diffusion process is shown in Fig. 4a. Following Hencky’s solution, the pressure difference across the membrane and the maximum deflection at the center of the suspended graphene hole follows a simple relation: Δ*P* *=* *k E t* ${d}_{2}^{3}/{r}^{4}$, where *k* is a dimensionless coefficient, *E* is Young’s modulus, *t* is the thickness of the graphene layer, *d*_2_ is the maximum deflection, and *r* is the circular radius of the suspended graphene region. Our experimental parameters for bubble filling are shown in Supplementary Fig. 17. Then, the samples were transferred to another chamber with NO_2_ gas molecules to tune the Fermi energy of graphene. HNO_3_ vapor was also used to dope the graphene and achieve higher carrier density. We verified that heating the doped samples produced a large decrease in doping.

### Near-field optical microscopy measurements

Near-field results were measured using a scattering SNOM setup (Neaspec GmbH) equipped with wavelength-tunable lasers (between 890 and 1600 cm^−1^). The spot sizes of the Mid-IR beam under the AFM tip were made ~30 μm in lateral size to cover the large area of suspended graphene, thus facilitating tip and edge launching of the GPs explored in this work. The probes were commercially available AFM tips metalized and having an apex radius of ~25 nm (Nanoworld). The tip-tapping frequency and amplitudes were ~270 kHz and ~30–50 nm, respectively. The near-field amplitude images were obtained from the third-order demodulated harmonic of the near-field amplitude, which resulted in massive suppression of background noise.

### Theoretical calculations and electromagnetic simulations

The dispersion relation of GPs can be obtained from the poles in the imaginary part of the Fresnel reflection coefficient ${r}_{p}\left(q,\omega \right)$, defined by the ratio between reflected and incident field amplitudes at the air/graphene/substrate interface, ${r}_{p}\left(q,\omega \right)=\frac{{\varepsilon }_{1}{k}_{0}\,-\,{\varepsilon }_{0}{k}_{1}\;+\;4\pi {k}_{0}{k}_{1}\sigma /\omega }{{\varepsilon }_{1}{k}_{0}\;+\;{\varepsilon }_{0}{k}_{1}\;+\;4\pi {k}_{0}{k}_{1}\sigma /\omega }$, where ${\varepsilon }_{0}$ is the dielectric constant of air, ${\varepsilon }_{1}$ is the complex dielectric function of SiO_2_, $\sigma =\sigma \left(q,\omega \right)$ is the in-plane optical conductivity of graphene, and *k*_0_ and *k*_1_ represent the out-of-plane light-wave-vector components in air and SiO_2_, respectively^27^. The permittivities of silicon dioxide and silicon (used later) at a light wavelength of 10.87 μm are ε(SiO_2_) = 3.8 and ε(Si) = 8.9, respectively^40^. For suspended graphene, we have ${r}_{p}\left(q,\omega \right)=\frac{4\pi {k}_{0}^{2}\sigma /\omega }{2{\varepsilon }_{0}{k}_{0}\;+\;4\pi {k}_{0}^{2}\sigma /\omega }$, In general, the graphene conductivity is a function of photon frequency $\omega$, Fermi energy ${E}_{F}$, inelastic relaxation time $\tau$, and temperature *T*. Under the conditions *k*_*B*_*T* ≪ ${{\hslash }}\omega$ and *k*_*B*_*T* ≪ |*E*_F_ | , the conductivity *σ* can be modeled in the local limit of the RPA^41^ and decomposed into intraband and interband contributions as ${\sigma ={\sigma }_{{intra}}+{\sigma }_{{inter}},{{{{{\rm{where}}}}}}\;\sigma }_{{intr}a}=\frac{{e}^{2}}{\pi {{{\hslash }}}^{2}}\frac{i{E}_{F}}{\omega \;+\;i{\tau }^{-1}}$, ${\sigma }_{{inter}}=i\frac{{e}^{2}}{4\pi {{\hslash }}}{{{{{\rm{ln}}}}}}\left(\frac{2\left|{E}_{F}\right|\;-\;{{\hslash }}\left(\omega \;+\;i{\tau }^{-1}\right)}{2\left|{E}_{F}\right|\;+\;{{\hslash }}\left(\omega \;+\;i{\tau }^{-1}\right)}\right),$ *e* is the electron charge, and ${{\hslash }}$ is the reduced Planck constant. The relaxation time $\tau =\mu {E}_{F}$/$e{v}_{F}^{2}$ depends on the graphene Fermi velocity ν_*F*_ = c/300 and the carrier mobility *μ*.

The simulated results shown in Figs. 1, 4, and Supplementary Fig. 5 were obtained using a Finite Elements Method package (COMSOL). In the simulation, the graphene layer was assigned a finite thickness *t*_g_ = 1 nm and an effective dielectric function ε = 1 + I $4\pi \sigma$/($\omega$*t*_g_). For the near-field image simulations in Fig. 1d and Supplementary Fig. 5, the graphene was modeled as a transition interface with the above dielectric properties. We approximated the tip by a vertical point dipole source featuring an oscillating dipole placed at a distance of 60 nm from the suspended graphene surface. The model was designed as an axisymmetric circular aperture structure, and we calculated the out-of-plane electric near-field component ${E}_{z}$ as a function of the dipole position along a radial line crossing the center of the circular suspended graphene region by exploiting the axisymmetry. The ${E}_{z}$ distribution was then expanded into a 2D axisymmetric plot. In the simulation, the contact angle *θ* was determined by the suspension height *d*_2_ through a function fitted from experimental measurements (Fig. 4b).

In the simulations of GP transmission in Fig. 4, the GPs were launched from a port to the left and then propagated along the graphene from the suspended region to the SiO_2_-supported area toward a port to the right. The simulation was performed on half of the suspended graphene region due to the symmetrical structure of the samples used in the experiments. The two numerical waveguide ports were placed at the ends of graphene to produce GPs. A GP impinging from the left at the interface could be reflected (GP moving to the left), transmitted (GP moving to the right), or out-coupled as a free-space IR photon. When the suspended graphene was above the substrate surface (i.e.*, d*_2_ > 0), the structure of suspended graphene was matched through a simple deflection profile as a result of the competition between the elastic energy of the deformed suspended graphene and the uniform force associated with gas pressure, as well as the adhesion energy (between graphene and the substrate), which can be fitted as $y(x)={d}_{2}(1-x^{2}/{a}^{2})$ with 95% confidence level^42^, where ${d}_{2}$ is the height of the center point of the suspended graphene to the substrate surface, and *a* = 1.25 μm is the radius of the hole. We set the center point of suspended graphene to $x$ = 0 (*x* being along the radial direction). The relationship between the contact angle *θ* and the height ${d}_{2}$ was extracted by taking the derivative at the edge of suspended graphene ($x$ = *a*): tan(*θ*) = 2${d}_{2}$/*a*, where *θ* is in the [0, π/2) range. Inelastic losses of GPs were not considered throughout the process, and the graphene thickness was assumed to be 1 nm. The reflectance *R* at the interface was defined as the ratio of the reflected GP energy (propagating to the left) to the incident GP energy (propagating from the left to the interface): $R=\frac{{|{E}_{R}|}^{2}}{{|{E}_{{in}}|}^{2}}$. We note that in our simulations the out-coupling channel produced only minor corrections.

## Supplementary information

Supplementary Information

## Data Availability

The data that support the findings of this study are available from the corresponding authors upon reasonable request.
