# Supplementary material for: Active control of micrometer plasmon propagation in suspended graphene
Source: Nat Commun. 2022 Mar 18;13:1465. doi: 10.1038/s41467-022-28786-8 (PMC8933486; doi:10.1038/s41467-022-28786-8)
Supplement: Supplementary file 1 — Supplementary Information [file 41467_2022_28786_MOESM1_ESM.pdf]

Supplementary Information for

Active control of micrometer plasmon propagation in suspended graphene

Hai Hu,[1, 2*] Renwen Yu,[3, 11] Hanchao Teng,[1, 2] Debo Hu,[1, 2] Na Chen,[1,2] Yunpeng Qu, [1, 2] Xiaoxia Yang,[1, 2] Xinzhong Chen,[5] A. S. McLeod,[6] Pablo Alonso-González,[7] Xiangdong Guo,[1, 2] Chi Li,[1, 2] Ziheng Yao,[5] Zhenjun Li,[1, 2] Jianing Chen,[8] Zhipei Sun,[9, 10] Mengkun Liu,[5] F. Javier García de Abajo,[3, 4*] Qing Dai[1, 2*]

1 CAS Key Laboratory of Nanophotonic Materials and Devices, CAS Key Laboratory of Standardization and Measurement for Nanotechnology, CAS Center for Excellence in Nanoscience, National Center for Nanoscience and Technology, Beijing 100190, P. R. China.
2 Center of Materials Science and Optoelectronics Engineering, University of Chinese Academy of Sciences, Beijing 100049, China.
3 ICFO-Institut de Ciencies Fotoniques, The Barcelona Institute of Science and Technology, 08860 Castelldefels (Barcelona), Spain.
4 ICREA-Institució Catalana de Recerca i Estudis Avançats, Passeig Lluís Companys 23, 08010 Barcelona, Spain.
5 Department of Physics and Astronomy, Stony Brook University, Stony Brook, New York 11794, USA.
6 Department of Physics, Columbia University, New York, NY, USA.
7 Departamento de Física, Universidad de Oviedo, Oviedo, Spain.
8 The Institute of Physics, Chinese Academy of Sciences, P.O. Box 603, Beijing, China.
9 Department of Electronics and Nanoengineering Aalto University Tietotie 3, FI-02150 Espoo, Finland.
10 QTF Centre of Excellence Department of Applied Physics Aalto University FI-00076 Aalto, Finland.
11 Present address: Department of Electrical Engineering, Ginzton Laboratory, Stanford University, Stanford, California 94305, United States

**e-mail*: daiq@nanoctr.cn, javier.garciadeabajo@nanophotonics.es, huh@nanoctr.cn

This PDF file includes:

Supplementary Figure 1. SEM, optical, and AFM images of suspended graphene samples.

Supplementary Figure 2. Tunable carrier densities of suspended graphene.

Supplementary Figure 3. Near-field images of suspended graphene without doping.

Supplementary Figure 4. Near-field images of suspended graphene acquired at different illumination frequencies.

Supplementary Figure 5. Selected rigorous simulations of near-field images.

Supplementary Note 1: Edge launching of suspended graphene plasmons.

Supplementary Figure 6. Tailored shape and size of nanoresonators based on suspended graphene plasmons.

Supplementary Figure 7. Schematic of the main four backscattered optical beams in near-field optical microscopy measurements.

Supplementary Note 2. Multi-beam interference model and calculation of quality factor.

Supplementary Figure 8. High-quality controllable plasmons in suspended graphene.

Supplementary Figure 9. Extraction of the quality factor of suspended graphene plasmons with different Fermi energies.

Supplementary Figure 10. Near-field images of graphene plasmons with different suspension heights.

Supplementary Figure 11. Quality factor as a function of the suspension height.

Supplementary Note 3. Graphene plasmon damping rate analysis.

Supplementary Figure 12. Method for calculating reflection.

Supplementary Figure 13. Simulation of plasmon reflectance as a function of contact angle and suspension height.

Supplementary Figure 14. Active control of graphene plasmon reflection by applying a gate voltage.

Supplementary Note 4. Active control of graphene plasmon reflection by applying a gate voltage.

Supplementary Figure 15. Tunable control of graphene plasmon reflection by varying the graphene Fermi energy.

Supplementary Note 5. Tunable control of graphene plasmon reflection by varying the graphene Fermi energy.

Supplementary Figure 16. Tunable control of graphene plasmon reflection by changing the excitation wavelength.

Supplementary Note 6. Tunable control of graphene plasmons reflection by changing the excitation wavelength.

Supplementary Figure 17. Experimental setup for bubble filling.

Supplementary References

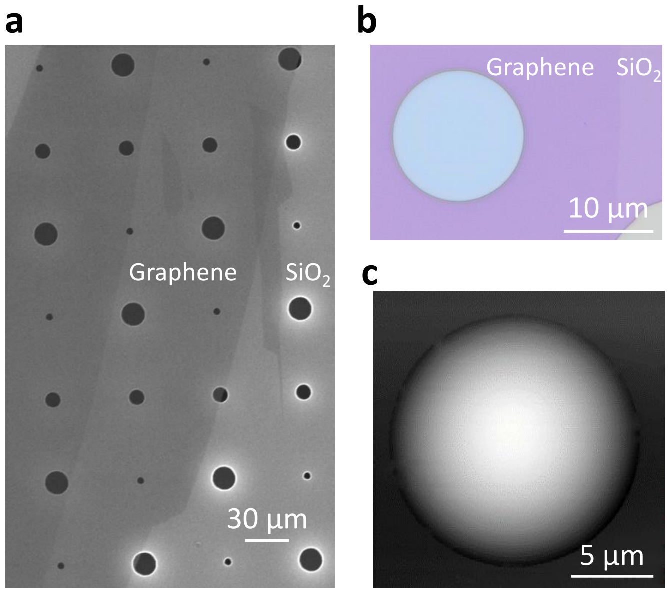

Supplementary Figure 1. SEM, optical, and AFM images of suspended graphene samples. (a) Typical SEM (scanning electronic microscope) images of our suspended graphene structures. **(b, c)** Optical (b) and topography micrographs (c) of the suspended graphene structure corresponding to the near-field image of Figure 1c.

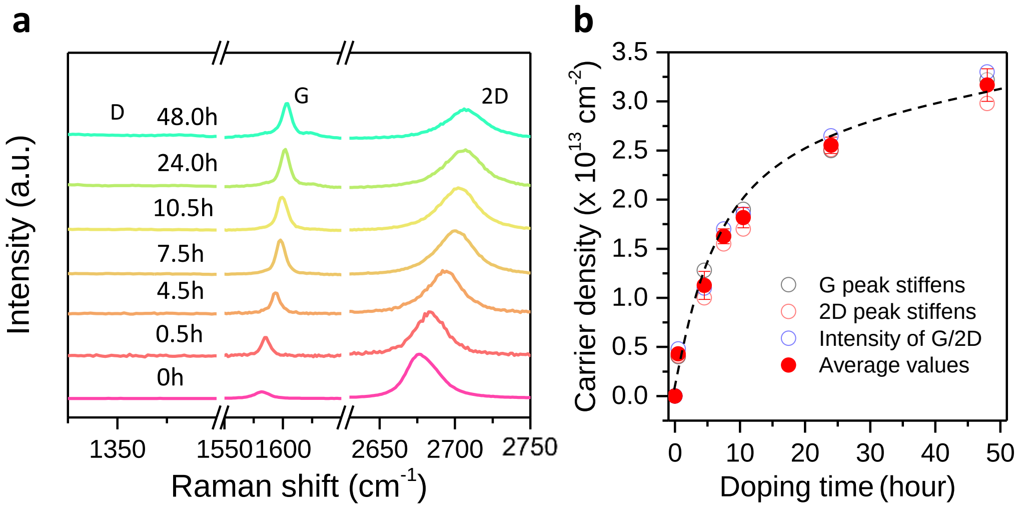

Supplementary Figure 2. Tunable carrier densities of suspended graphene. (a) Raman spectra of suspended graphene with different doping times. The concentration of NO_2 gas is 75% in an N_2 atmosphere. **(b)** Carrier densities of suspended graphene as a function of doping time**.** The carrier density is calculated from the G peak stiffening (grey circles), the 2D peak stiffening (red circles), and the intensity ratio I_G/I_{2D} (blue circles).[1] The solid red circles represent the average values of the above three calculation results. Symbols are obtained from experimental data whereas the curve is a guide to the eye.

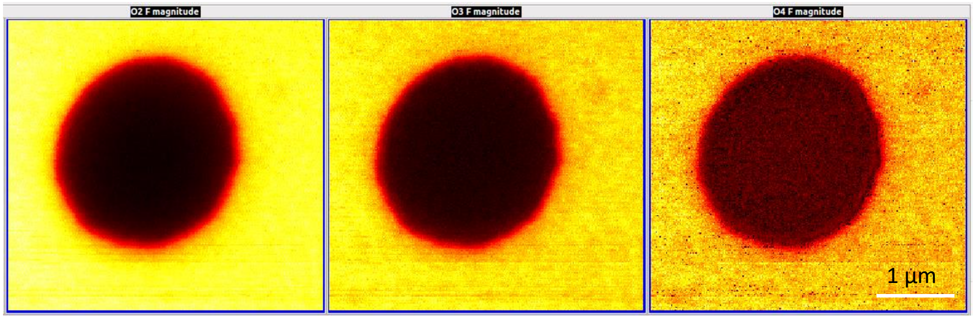

Supplementary Figure 3. Near-field images of suspended graphene without doping. The incident light wavelength is λ_0=10.87 µm. The scanning images are obtained from the second-, third-, and fourth-order demodulated harmonics of the near-field amplitude ***s***, from left to right. There are no fringes in these near-field images.

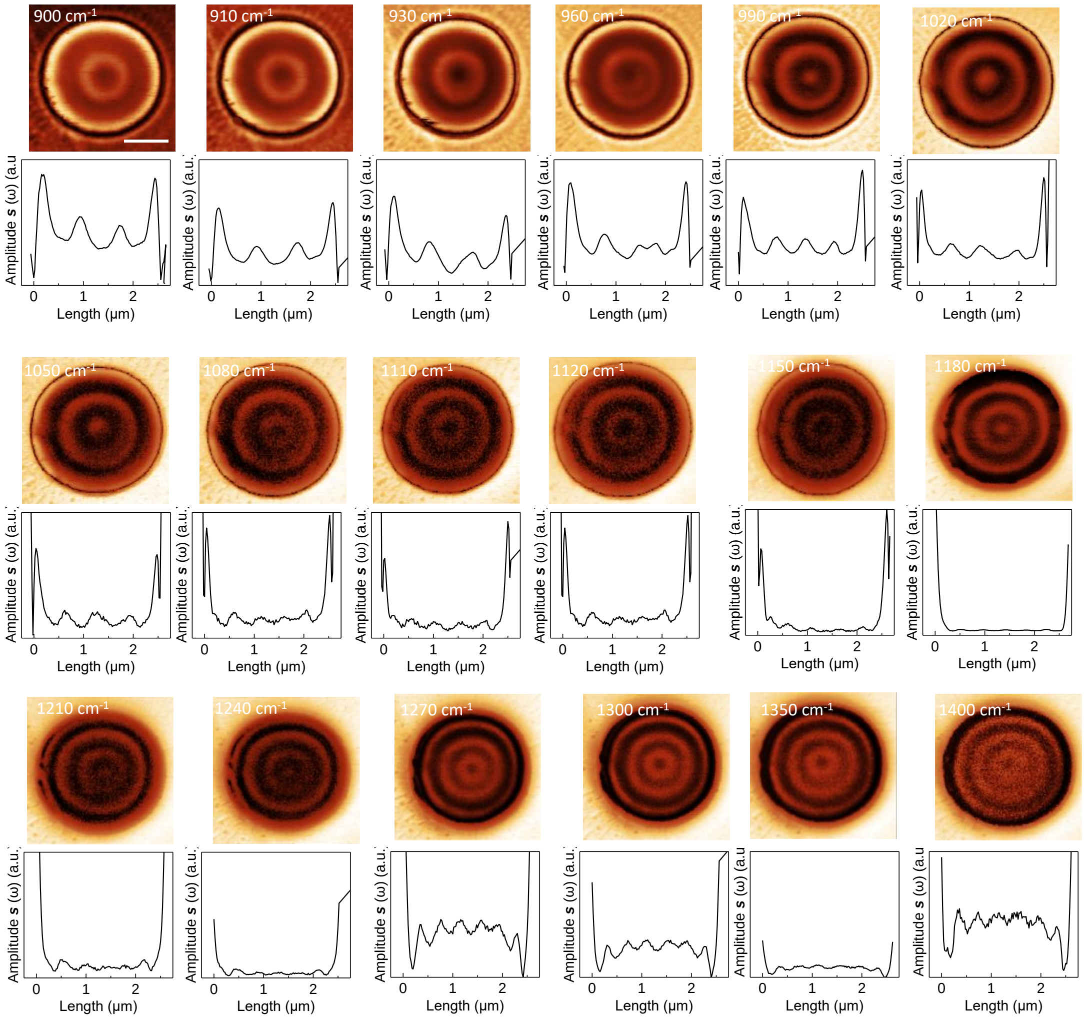

Supplementary Figure 4. Near-field images of suspended graphene acquired at different illumination frequencies. The diameter of the circular hole is Φ=2.5 µm. The graphene Fermi energy is ~0.90 eV. Line profiles below each image are taken from a horizontal line through the center of each near-field image. Scale bar indicates 1 µm.

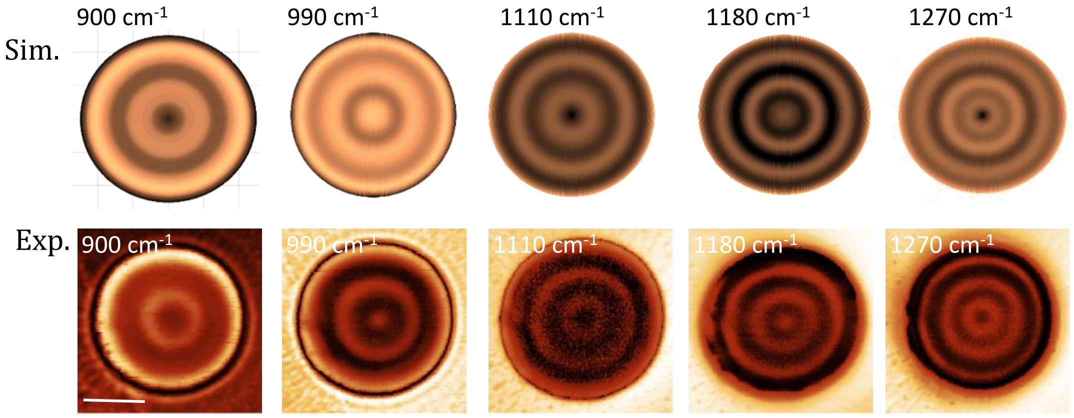

Supplementary Figure 5. Selected rigorous simulations of near-field images. Electromagnetic simulation of near-field amplitude images corresponding to the experimental results in Supplementary Figure 4. The upper part shows numerical modeling results. The lower part shows experimental images selected from Supplementary Figure 4. The scale bar indicates 1 μm.

Supplementary Note 1: Edge launching of suspended graphene plasmons.

For an external light plane wave incident on SiO_2 holes or dimples that are small compared to the wavelength, the response of the hole or dimple can be approximately captured by a set of induced dipoles distributed around its edge. In this work, these dipoles are used to couple light into optical modes of suspended graphene, and more precisely, into GPs. The polarizability α (relating the dipole to the external field) of a hole is typically proportional to a^3 (*i.e.*, α~a^3), where a is the hole radius. This scaling does not depend on the characteristics of the hole or dimple as long as it is small compared with the wavelength.[2, 3] Then, invoking the model of a circular array of dipole elements around the edge with a polarizability per unit length $d\alpha/dl$ to represent the response of the edge, the near field produced by $d\alpha/dl$ also grows with hole size, and so does the relative contribution of edge-launched plasmons compared with tip-launched plasmons, because the latter should depend less on hole size. This picture is consistent with our experimental results, showing that edge launching is more pronounced when the hole size increases.

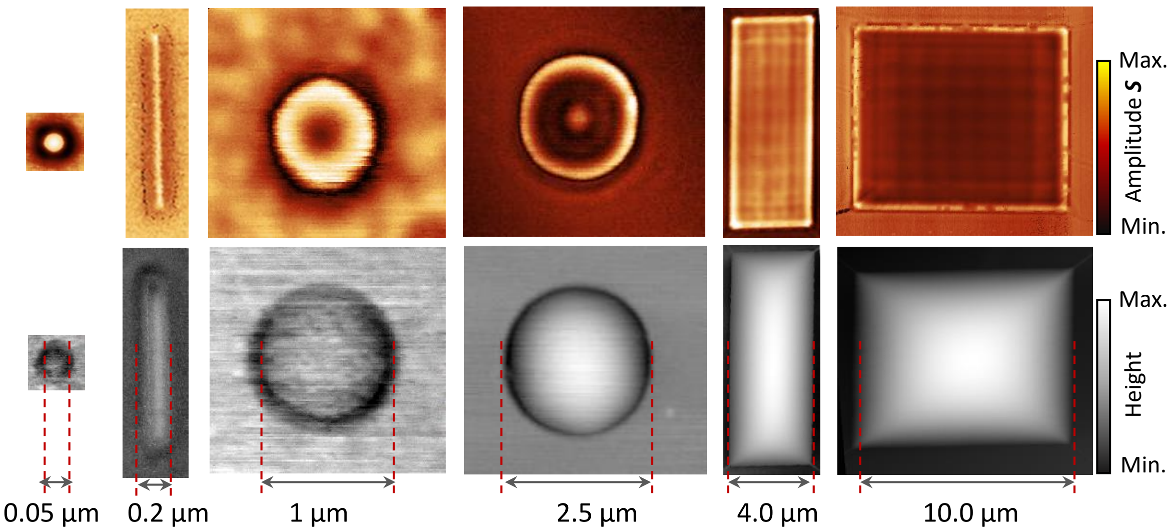

Supplementary Figure 6. Tailored shape and size of nanoresonators based on suspended graphene plasmons. Near-field amplitude images (upper panel) and corresponding AFM topography (lower panel) of nanoresonators are tailored in shape/size and feature suspended GPs.

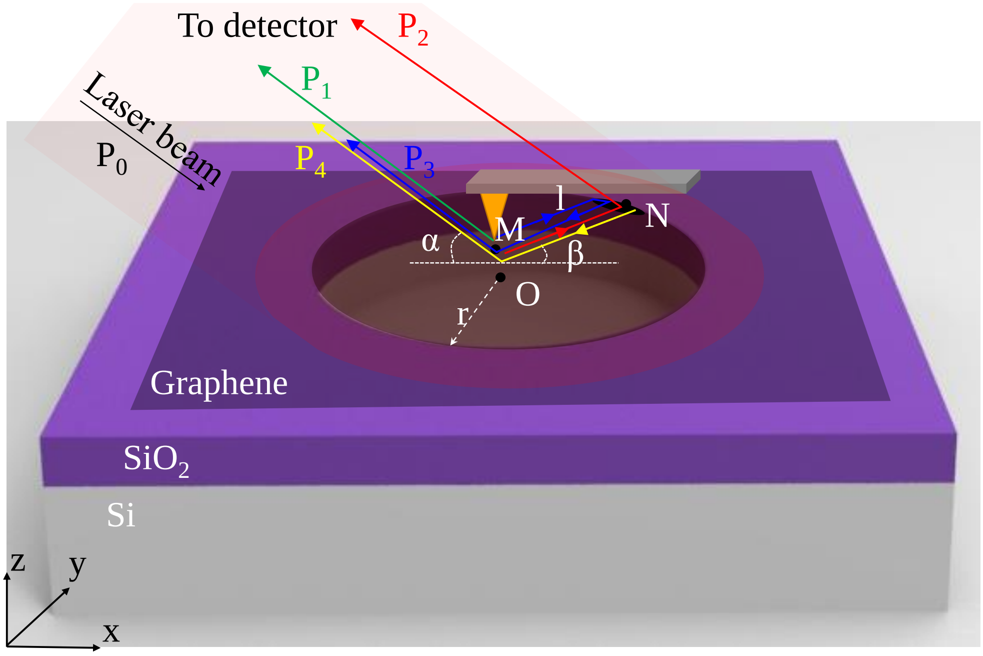

Supplementary Figure 7. Schematic of the main four backscattered optical beams (P_1-P_4) in near-field optical microscopy measurements. Point O is the center of the suspended graphene sample. M is an arbitrary point on the suspended graphene. N is a random point on the edge of the sample. The parameter r indicates the radius of the suspended graphene. α is the out-of-plane angle between the laser beam and the graphene surface. B is the angle subtended by the vector between M and N to the positive direction of the x axis. L represents

the tip-edge distance between points M and N.

Supplementary Note 2. Multi-beam interference model and calculation of quality factor.

To extract quality factors from the experimental data we closely follow a previously reported procedure based on a multi-beam interference model to analyze near-field signals.[4] As shown in Supplementary Figure 7, the incident laser beam (marked as P_0) illuminates the tip and sample, while a concave mirror is used to collect the backscattering photons from the system. The SNOM signals come from the following four main optical paths.[5] Firstly, incident photons are directly scattered by the tip back into the detector (marked as P_1); secondly, the tip-launched plasmons propagate away from the tip as circular waves and are then partially converted into photons at the edge of the hole, which is eventually scattered to the detector by the edge (marked as P_2); thirdly, the tip-launched plasmons propagate to the edge and then reflect the tip, where they are scattered toward the detector (marked as P_3); fourthly, the edge-launched plasmons propagate toward to the tip and are then scattered to the detector by the tip (marked as P_4). In the current study, the edge launching effect is important because the spot size of the mid-infrared laser is comparable to our large-size samples. If the sample size is larger than 20 μm, we actively defocus the laser beam to further expand the size of the light spot, thereby making the edge-launching more effective.

At an arbitrary point M of the suspended graphene, the near-field signal is the sum of the photon signals scattered back from this point and collected by the detector. Then, the total optical signal $|P|$ is given by

$$|P| = \left|\sum_{j=1}^{4} P_j\right|. \quad \text{(Supplementary Equation 1)}$$

The wave function of each optical path can be conveniently written as

$$P_j = A_j e^{i\varphi_j}, \quad j = 1 - 4, \quad \text{(Supplementary Equation 2)}$$

where A_j is the amplitude and φ_j the phase of path j. We set the phase of P_1 to 0. Then, P_2, P_3, and P_4 have a phase delay relative to P_1 that depends on the tip-edge distance (marked as l) between points M and N. The phase of each path is given by

$$\begin{cases} \varphi_1 = 0, \\ \varphi_2 = \varphi_4 = ((k_0 \cos\alpha)\cos\beta + q)l, \\ \varphi_3 = 2ql, \end{cases} \quad \text{(Supplementary Equation 3)}$$

where $k_0 = 2\pi/\lambda_0$ is the free-space wave vector, q is the wave vector of the excited GPs, α is the out-of-plane angle between the laser beam and the graphene surface, and β is the angle formed by the vector joining M and N relative to the positive direction of the x axis. The amplitude A of each path is related to the plasmon scattering rate of the tip (η_t), the edge scattering rate (η_e), the edge reflectivity (η_R), and the plasmon damping. The loss of plasmons from the tip is caused by a combination of damping (e.g., inelastic scattering by charge-impurities and defects, $\sim e^{-l/a}$, where a is the decay length) and circular-wave geometrical spreading ($\sim 1/\sqrt{l}$).[6]

The amplitude of each path can then be expressed as

$$\begin{cases} A_1 = \eta_s A_0, \\ A_2 = \eta_t \eta_e A_0 l^{-1/2} e^{-l/a}, \\ A_3 = \eta_t^2 \eta_R A_0 (2l)^{-1/2} e^{-2l/a}, \\ A_4 = \eta_e \eta_t A_0 l^{-1/2} e^{-l/a}, \end{cases} \qquad \text{(Supplementary Equation 4)}$$

where A_0 is the amplitude of the incident laser beam. Based on Supplementary Equations (1-S), the total optical signal $|P|$ of the entire suspended graphene is given by

$$|P| = \left| \eta_t A_0 \left(\frac{\eta_s}{\eta_t} + 2\eta_e \int_0^{2\pi} l^{\frac{1}{2}} e^{-\frac{l}{a}} e^{i(k_\perp \cos\beta + q)l} \mathrm{d}\beta + \eta_t \eta_R \int_0^{2\pi} \sqrt{2} l^{\frac{1}{2}} e^{-\frac{2l}{a}} e^{i2ql} \mathrm{d}\beta \right) \right|.$$

(Supplementary Equation 5)

With this multi-beam interference model, we can fit the decay length a, and from here the quality factor can be defined as Q=2π a/λ_p. To properly exclude the influence of the background signal, each curve was fitted twice. Accurately fitting the front part with a strong signal (Q_1 in Supplementary Figure 8a) and the back part with a weak signal (Q_2 in Supplementary Figure 8b), we then calculate the average Q=(1/2)(Q_1+Q_2).

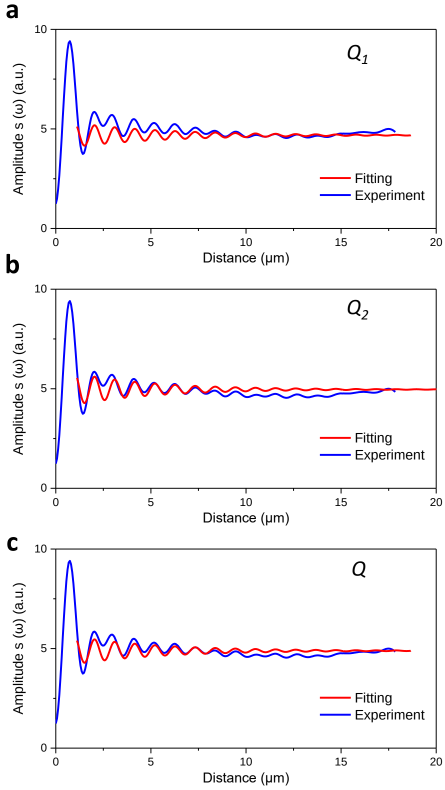

Supplementary Figure 8. High-quality controllable plasmons in suspended graphene. Blue curves are extracted from typical experimental near-field images of suspended graphene. Red curves represent the fitting results.

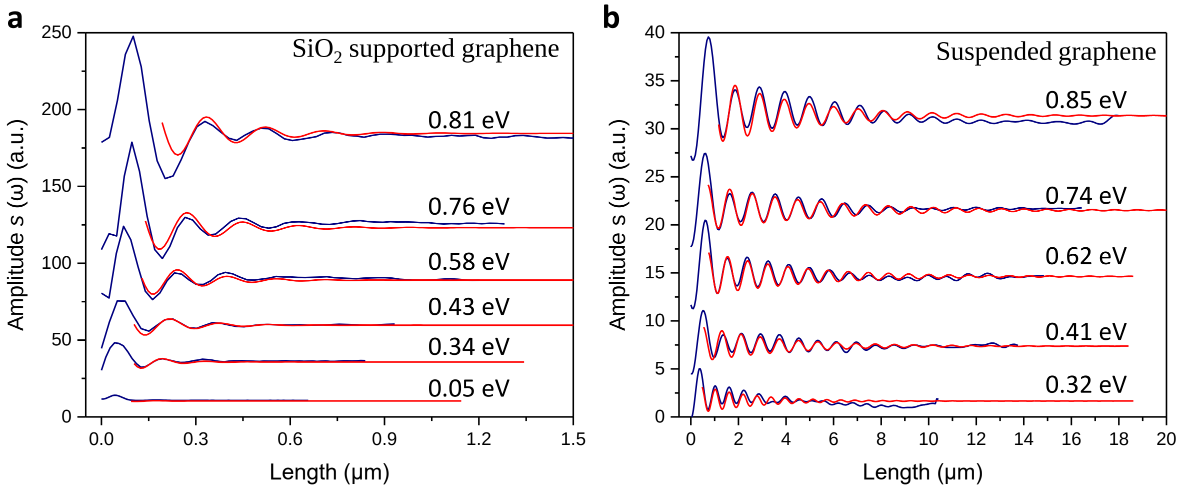

Supplementary Figure 9. Extraction of the quality factor of suspended graphene plasmons with different Fermi energies. Line profiles correspond to the near-field images of Figures 2a, b in the manuscript.

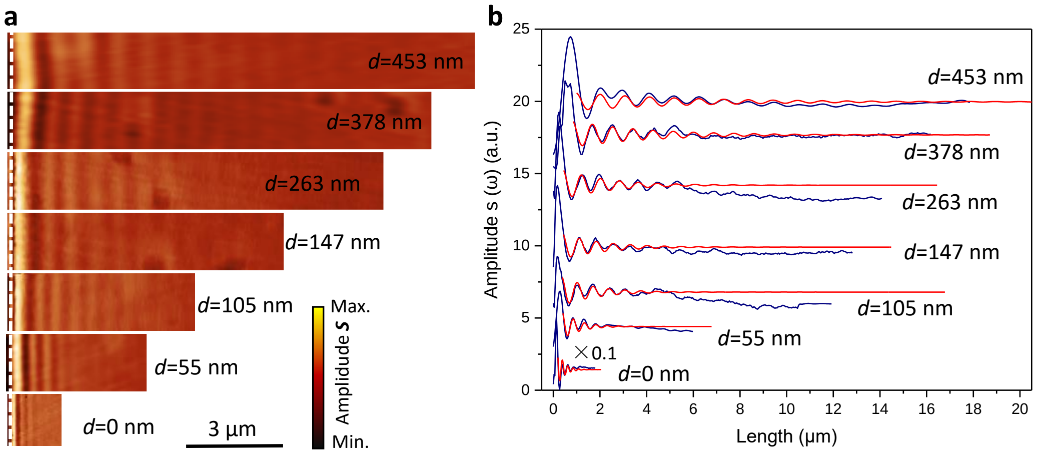

Supplementary Figure 10. Near-field images of graphene plasmons with different suspension heights. (a) Near-field amplitude images of suspended graphene with different suspension heights *d*. **(b)** Line profiles corresponding to the near-field images of Supplementary Figure 10a. Blue curves represent the experimental data and red curves stand for the fitting results. Three different samples were prepared to cover a wide range of suspension heights. Sample #1 was used for *d*=0, with the graphene directly in contact with the substrate. Sample #2 was used for *d*=55 nm, 105 nm, and 147 nm; the depth and diameter of the hole were 100 nm and 20 μm, respectively. Sample #3 was used for *d*=263 nm, 378 nm, and 453 nm; the depth and diameter of the hole were 200 nm and 30 μm, respectively.

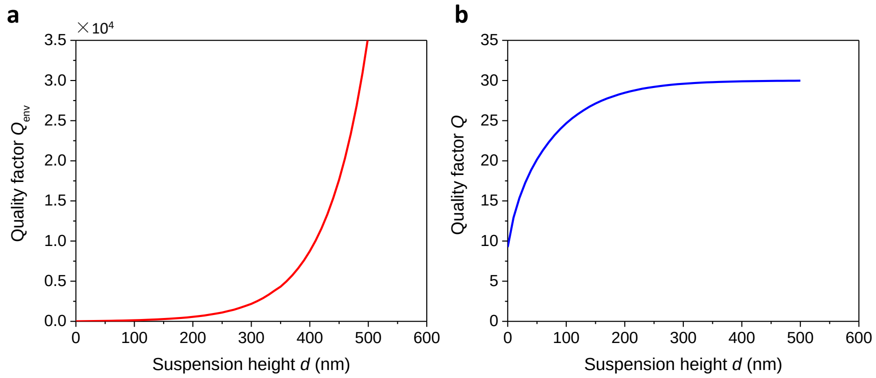

Supplementary Figure 11. Quality factor as a function of the suspension height. (a) Q_{env} as a function of the suspension height d. **(b)** Q as a function of the suspended height d with Q_{gra} assumed to be 30.

Supplementary Note 3. Graphene plasmon damping rate analysis.

The inverse of the quality factor Q can be split into two additive parts

$$Q^{-1} \approx \frac{\sigma_1}{\sigma_2} + \frac{\kappa_2}{\kappa_1} = Q_{gra}^{-1} + Q_{env}^{-1}, \qquad \text{(Supplementary Equation 6)}$$

where the first term on the right-hand side, $Q_{gra}^{-1} \approx \frac{\sigma_1}{\sigma_2}$, represents the intrinsic damping from graphene with the graphene optical conductivity $\sigma \approx \sigma_1 + i\sigma_2$, whereas the second term, $Q_{env}^{-1} = \frac{\kappa_2}{\kappa_1}$, describes the dielectric losses from the environment with an effective dielectric permittivity $\kappa = \kappa_1 + i\kappa_2$. We use the following separation-dependent expression for the latter:

$$\kappa\,(q,\omega) = \frac{1+\epsilon_{SiO_2}}{1+\epsilon_{SiO_2}+(1-\epsilon_{SiO_2})e^{-2qd}}\;, \qquad \text{(Supplementary Equation 7)}$$

where ϵ_{SiO_2} is the permittivity of SiO_2, q is the plasmon wave vector, and d is the suspended height. In the $d = 0$ limit, we recover the well-known result $\kappa = (1+\epsilon_{SiO_2})/2$. In Supplementary Figure 11a, Q_{env} is calculated from Eq. S6 as a function of the suspended height d. After assuming $Q_{gra} = 30$, we obtain the total quality factor Q shown in Supplementary Figure 11b.

Multiple channels are involved in graphene plasmon damping, including losses due to scattering by charged impurities, phonons, and electron-electron (e-e) collisions, as well as dielectric losses. In our suspended graphene samples, the contribution of optical phonons to scattering is weak because of their high energy.[6, 7] The contribution of acoustic phonons to plasmon losses is associated with scattering between electrons and low-energy lattice vibrations in graphene, so it is weakly dependent on carrier density.[8] Charged impurity scattering leads to strongly reduced damping when increasing the carrier density, owing to the enhanced electrostatic screening.[8] Therefore, the main loss mechanisms of suspended graphene plasmons at room temperature are charged impurity scattering and acoustic phonon scattering.

Our system is very different from previous studies (*e.g.*, BN encapsulated graphene[7, 8]) due to the chemical doping scheme used here, which can potentially introduce additional impurity

scattering (and even modify the phonon bands of graphene [9]). Therefore, it is difficult to estimate Q_{gra} and its doping dependence. If the additionally charged impurity scattering becomes a dominant loss pathway, increasing the doping level will improve the quality factor because of the increase in screening.

We note that the dependence of the quality factor on the suspension height does not quickly reach saturation (Figure 3d of main text) because graphene is not entirely horizontal. For Sample 2, with a low suspension height, graphene adopts a parabolic profile at the pressure inside the graphene bubble. Therefore, starting from the boundary, as the downward slope of the graphene is closer to the substrate, the loss of the graphene plasmon will increase. As a result, the quality factors at d= 55 nm, 105 nm, and 147 nm should be underestimated, so the curve does not quickly saturate.

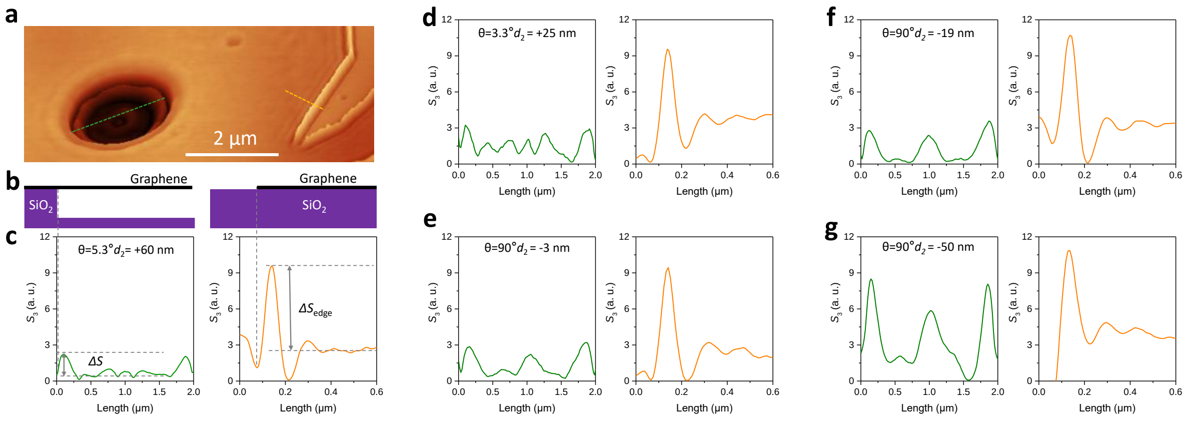

Supplementary Figure 12. Method for calculating reflection. (**a**) Typical near-field IR image of suspended graphene. The incident light wavelength is λ_0= 10.87 µm (920 cm^{-1}), and the graphene Fermi energy is 0.40±0.03 eV. The experimental near-field amplitude profiles across the air-dielectric interface and natural graphene edge are indicated by green and orange dashed lines, respectively. The scale bar indicates 2 µm. (**b**) illustration of a cross-sectional side view of the air-dielectric interface and natural graphene edge. (**c**) Experimental near-field amplitude profiles across a graphene air-dielectric interface (left) and natural edges (right), extracted from the near-field IR images of (a). The horizontal dashed lines mark the averaged signal on graphene. The vertical dashed lines mark the positions of the graphene air-dielectric interface and natural edges, respectively. (**d-g**) Experimental amplitude profiles corresponding to the near-field images of Figure 4c in the manuscript.

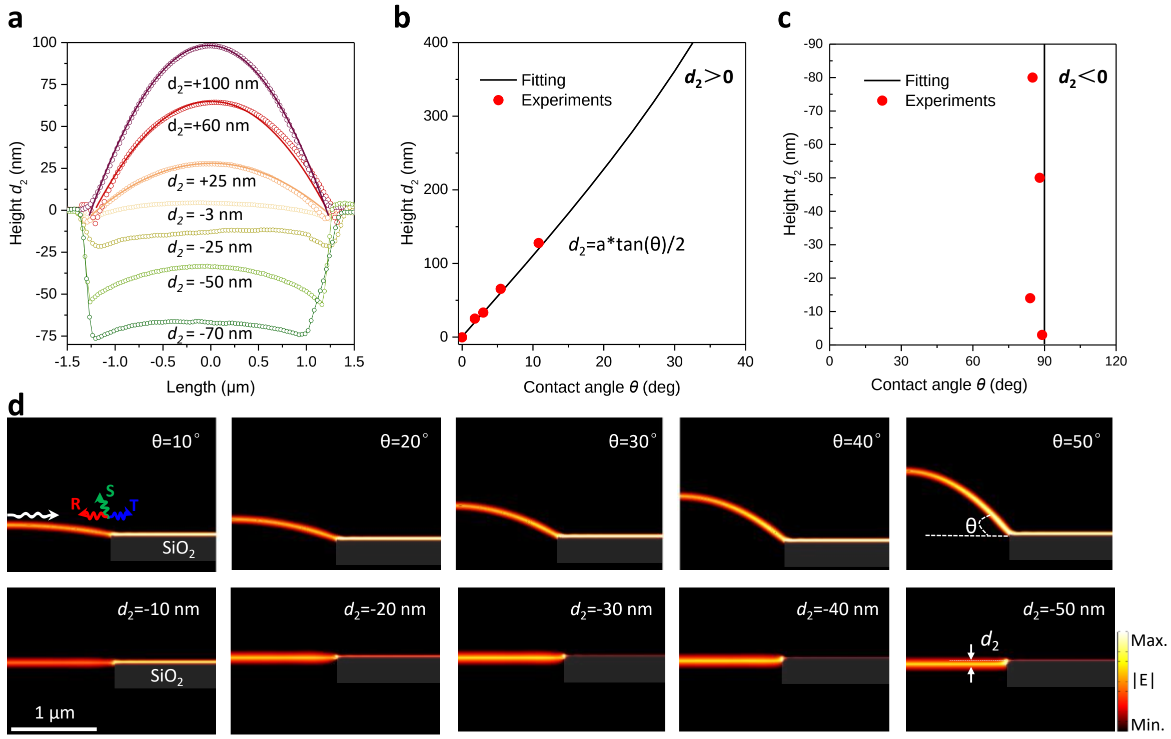

Supplementary Figure 13. Simulation of plasmon reflectance as a function of contact angle and suspension height. (a) Height profiles of suspended graphene bubbles, shown in Figure 4b in the main text. Symbols and curves indicate experimental data and the corresponding fitted parabolic functions when d_2>0. **(b)** and **(c)** Relation between d_2 and θ when the graphene is above the substrate surface (*i.e.* d_2>0) and below the substrate surface (*i.e.,* d_2<0) in the suspended region, respectively. The relations are fitted to experimental data with 95% confidence intervals. **(d)** Simulated spatial distributions of the amplitude of the electric field as plasmons propagate from the suspended graphene region into the area supported by the SiO_2 substrate with different contact angles θ and step heights d_2, respectively. Here, we take E_F=0.4 eV and λ_0=10.87 μm (920 cm^{-1}). White, red, and blue arrows represent incident, reflected, and transmitted plasmons, respectively. The green arrow stands for plasmon scattering (out-coupling) into radiation.

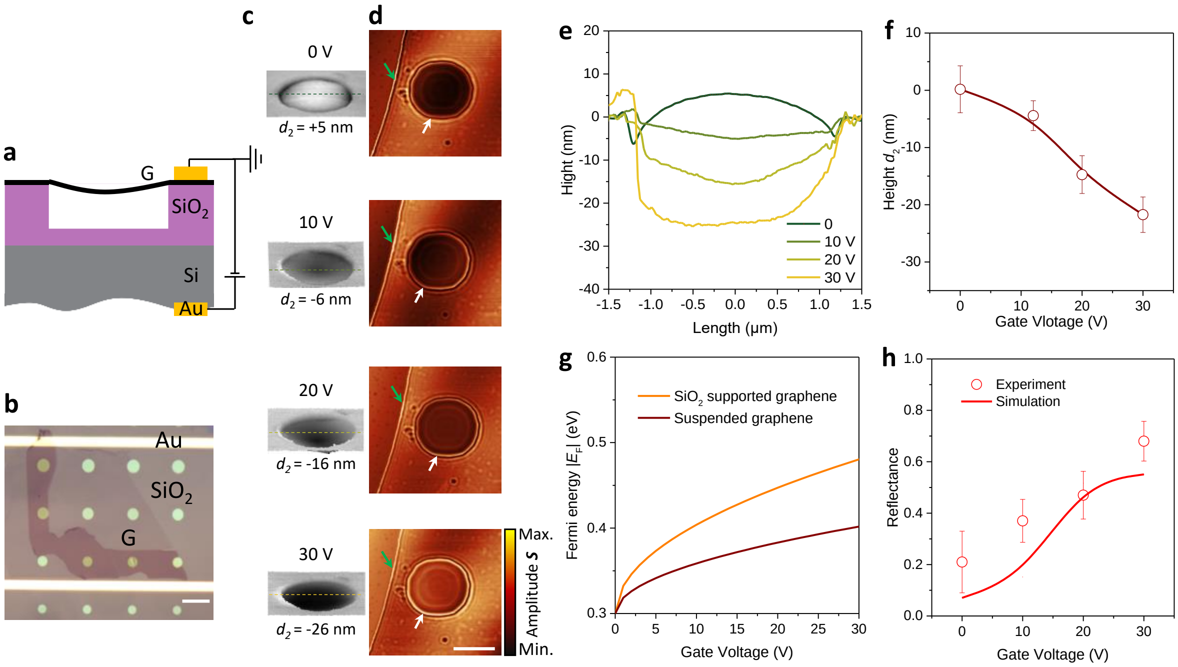

Supplementary Figure 14. Active control of graphene plasmon reflection by applying a gate voltage. (a) Illustration of a cross-sectional side view of a gate-controllable suspended graphene device. **(b)** Optical image of a typical suspended graphene device with gold electrodes. The scale bar indicates 5 μm. **(c)** AFM topography images of suspended graphene with different heights of the graphene bubble d_2, obtained by controlling gate voltages for fixed hole depth d_1=300 nm (see definitions in the inset of Figure 3b). **(d)** Near-field IR images of suspended graphene, taken simultaneously with the AFM topography. The incident light wavelength is λ_0= 10.87 μm (920 cm^{-1}). The bright fringes at the air-dielectric interface and natural graphene edge are indicated by white and green arrows, respectively. The scale bar indicates 2.0 μm. **(e)** Height profiles of suspended graphene bubbles. **(f)** Relation between d_2 and gate voltages, extracted from the height profiles in (e). **(g)** Graphene Fermi energy shifts with gate voltages for suspended graphene and SiO_2 supported graphene. **(h)** Plasmon reflectance as a function of gate voltages. Symbols are obtained from experimental data of (d), while the curve is a guide to the eyes.

Supplementary Note 4. Active control of graphene plasmon reflection by applying a gate voltage.

To gain dynamic control over GP reflection and further prove the terms we used, we have carried out a series of new experiments to quickly and effectively control plasmon transmission at the dielectric boundary by applying a gate voltage, varying the graphene Fermi energy, and changing the incident wavelength (Supplementary Figure 14-16). Especially through the gate voltage, the graphene can be electrostatically deformed and doped at the same time to obtain the sought-after dynamic control of GP transmission (Supplementary Figure 14).

When a transverse electric field is applied across the suspended graphene, a moment of force acting on the graphene induces electric polarization. As a result, the graphene becomes

electrically charged and is attracted to the counter electrode. Because it can be regarded as a thin atomic membrane with low lateral stiffness, suspended graphene can be an ideal material for electromechanical devices to control morphology. Supplementary Figures 14 a,b shows a schematic and an optical image of a gate controllable suspended graphene device. Note that our suspended graphene is doped to 0.3 eV by NO_2 molecules before adding a gate voltage. Since the molecules are always adsorbed on the graphene surface, the entire system is maintained electrically neutral. Supplementary Figure 14c shows an AFM image of a circular suspended bubble, which demonstrates precise manipulation of suspended graphene morphology via electrostatic control. The graphene membrane is gradually sucked into the holes with a vertical displacement. The height of the suspended graphene relative to the substrate can be adjusted from +5 nm to -26 nm. Supplementary Figure 14d shows a near-field optical image corresponding to the AFM image. The natural boundary of graphene is also introduced to facilitate the direct calculation of the reflection at the dielectric boundary. How the morphology of graphene affects the impedance mismatch and reflection of plasmons has been discussed in detail in the manuscript.

Since the gate voltage applied by the suspended graphene and SiO_2 supported graphene is the same, the graphene's carrier concentration on both sides strongly depends on the effective capacitance contributed by the dielectric constant of air and SiO_2. As shown in Supplementary Figure 14g, when the gate voltage is tuned from 0 to 30 V, the change of $|E_F|$ shifts from about 0.3 to 0.5 eV for SiO_2 supported graphene. This shift of $|E_F|$ is larger than the change for suspended graphene, from about 0.3 to 0.4 eV under the same gate voltages. Therefore, with a continuous increase of the gate voltage, the difference of height and the Fermi level on both sides can be exacerbated at the same time, which further promotes the reflection of plasmons at the boundary. As shown in Supplementary Figure 14h, the reflection of plasmons at the dielectric boundary increases with increasing gate voltages, from 23% to 70%.

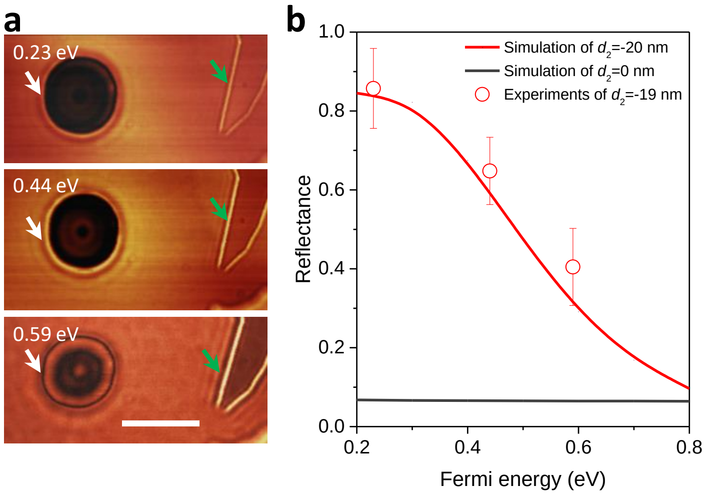

Supplementary Figure 15. Tunable control of graphene plasmon reflection by varying the graphene Fermi energy. (a) Near-field infrared image of suspended graphene with different Fermi energies is controlled by chemical doping. The bright fringes at the air-dielectric interface and natural graphene edge are indicated by green and white arrows, respectively. The scale bar indicates 2.5 μm. **(b)** Plasmon reflectance as a function of Fermi energy. Symbols are

obtained from experimental data of (a), while curves are from the simulation.

Supplementary Note 5. Tunable control of graphene plasmon reflection by varying the graphene Fermi energy.

An essential feature of graphene over metals as plasmonic materials is the ability to change the carrier concentrations to modulate the plasmon performance. Therefore, we explore the corresponding plasmons switching effect by adjusting the graphene carrier concentration. This facilitates the mutual compatibility of our plasmons switch and other electronic devices. Supplementary Figure 15a shows a near-field infrared image of suspended graphene with different carrier densities (corresponding to a Fermi energy varying from ~0.2 to 0.6 eV). As the Fermi level increases, the fringes at the dielectric boundary and the natural edge become brighter. This indicates that the strength of the plasmon resonance is enhanced with increasing doping concentration. Besides, the number of fringes of the suspended graphene region is also reduced because the graphene plasmon's wavelength is increased with increasing doping concentration. We calculated the reflection as a function of graphene Fermi level. As illustrated in Supplementary Figure 15b, the reflectance of plasmons at the dielectric boundary decreases with increasing doping levels, from 85% to 42%. A longer wavelength can enhance plasmon diffraction effects to transmit boundary regions.

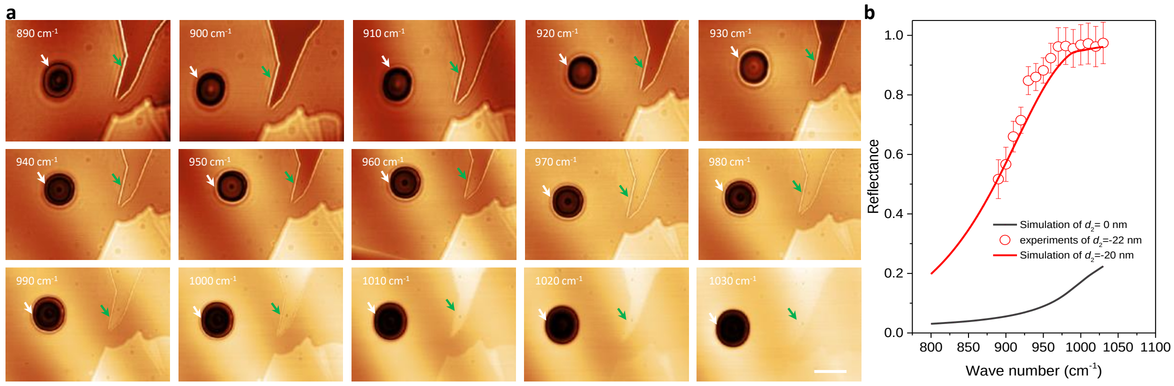

Supplementary Figure 16. Tunable control of graphene plasmon reflection by changing the excitation wavelength. (a) Near-field infrared images of suspended graphene with different excitation wavelengths. The bright fringes at the air-dielectric interface and natural graphene edge are indicated by white and green arrows, respectively. The scale bar indicates 2.5 μm. **(b)** Plasmon reflectance as a function of excitation wavelength. Symbols are obtained from experimental data of (a), while curves are from the simulation.

Supplementary Note 6. Tunable control of graphene plasmon reflection by changing the excitation wavelength.

It is also possible to achieve *in-situ* modulation of reflection by changing the excitation wavelength. As shown in Supplementary Figure 16a the plasmon wavelength is decreased with the increase of excitation energy, but the reflectance gradually increases as the excitation energy gets closer to the surface optical phonons of SiO_2. Besides, the background of the near-field image gradually becomes more intense near these phonon frequencies. Compared with Fermi energy modulation, changing the incident wavelength is more effective, and the regulation range is extended from 51.6% to 97.4%. It is worth noting that when the graphene bubble is nearly flat (d_2=0, *i.e.*, co-planar with the substrate surface), both by changing the excitation wavelength (Supplementary Figure 16b) or the Fermi energy (Supplementary Figure 15b), the tunability of the plasmons at the dielectric boundary is very weak.

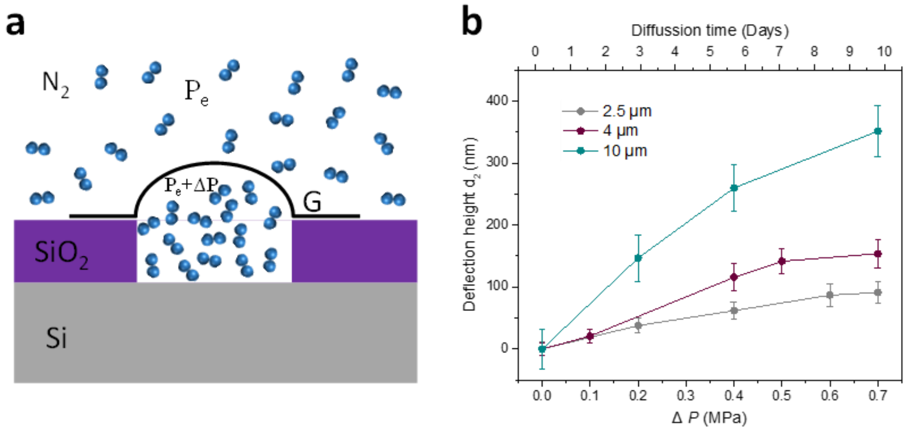

Supplementary Figure 17. Experimental setup for bubble filling. (a) Schematic of gas diffusion into a graphene bubble from the chamber when applying very high pressure (P_a). P_e indicates the external atmospheric pressure. **(b)** Experimental parameters for bubble filling.

Supplementary References

1. Das, A. *et al*. Monitoring dopants by Raman scattering in an electrochemically top-gated graphene transistor. *Nature Nanotech.* **3**, 210-215 (2008).
2. Bethe, H.A. Theory of diffraction by small holes. *Phys. Rev.* **66**, 163 (1944).
3. De Abajo, F.G. Colloquium: Light scattering by particle and hole arrays. *Rev. Mod. Phys.* **79**, 1267 (2007).
4. Liao, B. *et al*. A Multibeam Interference Model for Analyzing Complex Near-Field Images of Polaritons in 2D van der Waals Microstructures. *Adv. Funct. Mater.* **29**, 1904662 (2019).
5. Hu, F. *et al*. Imaging exciton–polariton transport in $MoSe_2$ waveguides. *Nature Photon.* **11**, 356-360 (2017).
6. Principi, A. et al. Plasmon losses due to electron-phonon scattering: The case of graphene encapsulated in hexagonal boron nitride. *Phys. Rev. B* **90**, 165408 (2014).
7. Ni, G.X. *et al*. Fundamental limits to graphene plasmonics. *Nature* **557**, 530-533 (2018).
8. Woessner, A. *et al*. Highly confined low-loss plasmons in graphene-boron nitride heterostructures. *Nature Mater.* **14**, 421-425 (2015).
9. Novko, D. Dopant-induced plasmon decay in graphene. *Nano Lett.* **17**, 6991-6996 (2017).
